# Screening for Asymptomatic Osteonecrosis of the Hip in Systemic Lupus Erythematous: A Systematic Review and Meta-Analysis of MRI-Based Prevalence

**DOI:** 10.3390/diagnostics14030279

**Published:** 2024-01-27

**Authors:** Hamza A. Ibad, Elena Ghotbi, Arta Kasaeian, Adam S. Levin, Lynne C. Jones, Yoshimi Anzai, Maryam Soltanolkotabi, Neena Kapoor, Pamela T. Johnson, Shadpour Demehri

**Affiliations:** 1 The Russell H. Morgan Department of Radiology and Radiological Science, Johns Hopkins University School of Medicine, Baltimore, MD 21287, USA; 2 Department of Orthopedic Surgery, The Johns Hopkins University, Baltimore, MD 21287, USA; alevin25@jhmi.edu; 3 Center for Osteonecrosis Research and Education, Department of Orthopedic Surgery, Johns Hopkins University School of Medicine, Baltimore, MD 21287, USA; 4 Department of Radiology and Imaging Sciences, University of Utah, Salt Lake City, UT 84132, USA; yoshimi.anzai@hsc.utah.edu (Y.A.);; 5 Department of Radiology, Brigham and Women’s Hospital, Harvard Medical School, Boston, MA 02115, USA

**Keywords:** systemic lupus erythematosus, magnetic resonance imaging, osteonecrosis, hip joint

## Abstract

*Objective*. This paper aims to estimate asymptomatic hip osteonecrosis prevalence in SLE patients using MRI examination and to determine the prevalence among higher risk subpopulations. *Materials and Methods*. PubMed, Embase, Cochrane, and SCOPUS were searched from inception to May 9th, 2023. Studies on patients who were clinically diagnosed with systemic lupus erythematosus without reported symptoms attributable to hip osteonecrosis were included. Two independent reviewers extracted data and assessed the risk of bias. Data collected from each study include the study year, the number of hips screened, the number of hips with osteonecrosis, demographics, laboratory data, medications, follow-up time, radiological protocols, and MRI-based osteonecrosis detection and grading criteria. *Results*. Eleven eligible studies including 503 participants (15–35 years old; 74–100% female) with SLE were identified. Significant risk of bias was determined in one study. The overall prevalence of osteonecrosis of the hip was found to be 14% (184/1006 hip joints, 95% confidence interval: 7–22%, number needed to scan: 7.1). SLE patients who received corticosteroid treatment had a higher prevalence of asymptomatic hip osteonecrosis (18%) compared to non-corticosteroid users (0%, *p*-value < 0.01). Additionally, meta-regression results revealed that daily corticosteroid dose was associated with increased prevalence of asymptomatic osteonecrosis (0.5%/milligram, *p*-value < 0.01). *Conclusions*. The high prevalence of asymptomatic hip osteonecrosis in SLE patients raises concerns about the timeliness of interventions. The limitations of this study include a relatively low number of identified studies; and one study lacked full-text availability.

## 1. Introduction

Systemic lupus erythematosus (SLE) is a multi-systemic chronic disease affecting 72.8 per 100,000 individuals in the US [1]. It is estimated that individuals with SLE incur upwards of USD 10,000 in annual healthcare costs and utilization in the US [2]. Thus, implementing cost-saving strategies for SLE management is crucial, focusing on timely prevention of complications like osteonecrosis [3]. Risk factors for osteonecrosis in SLE include disease duration, high low-density lipoprotein cholesterol, antiphospholipid antibody positivity, and corticosteroid use [4]. Among patients with a history of systemic corticosteroid administration, SLE has been reported as the most frequent underlying disease associated with hip osteonecrosis [5]. However, osteonecrosis can also occur in SLE patients who are not receiving corticosteroids [4,6]. Among SLE patients, osteonecrosis is predominantly seen in the femoral head (73%), followed by the shoulder (27%), knee (27%), and metatarsal head (9%) [7].

Hip osteonecrosis is commonly associated with pain and reduced range of motion [8]. Early-stage osteonecrosis is typically managed conservatively with limited weight-bearing alongside shockwave therapy, electromagnetic fields, hyperbaric oxygen, bisphosphonates, or anticoagulants [9,10]. Surgical options include emerging core decompression, injection of autologous stem cells, and various osteotomies. Given the significant proportion (up to 56%) of asymptomatic hip osteonecrosis cases that progress to a symptomatic stage [11], timely diagnosis is crucial to preserve the joint and delay total hip replacement [12,13], especially considering that osteonecrosis predominantly affects young individuals (~39 years of age from all causes [9]). In addition, prior reports suggest that once a patient develops symptoms, the onset of joint collapse can be within 1 year [14,15,16]. Thus, detection of osteonecrosis in high-risk SLE patients can play a crucial role in preserving the integrity of the native joint and avoiding or delaying downhill costs and morbidities [17]. 

Current Appropriateness Criteria^®^ by the American College of Radiology recommends radiographs as the initial imaging modality for individuals with clinical suspicion, e.g., pain [18]. However, in the pre-symptomatic stages of osteonecrosis, radiographs may not reveal any apparent abnormalities [14]. As the condition progresses, radiographs may show a characteristic crescent sign, indicating subchondral fracture and degenerative changes [19]. To diagnose osteonecrosis at an early phase, before the typical radiographic changes and patient-reported pain occur, MRI is considered the “gold standard” diagnostic tool [18,19]. In early, asymptomatic stages of the disease, MRI can reveal a band or ring-like zone of decreased signal intensity on T1 weighted images or a ‘double-line’ sign on T2 weighted images [20].

In a prior meta-analysis, particular attention was given to the prevalence of osteonecrosis in both symptomatic and asymptomatic patients [21]. We aim to contribute to these findings by focusing on asymptomatic cases at the level of the hip joint. Thus, this study aimed to determine the prevalence of asymptomatic osteonecrosis in the hip joints of individuals with SLE as detected by MRI and subsequent subgroup and meta-regression analyses.

## 2. Methods

### 2.1. Data Sources

Our review was structured according to the preferred reporting items for systematic reviews and meta-analyses [22] and the meta-analyses of observational studies in epidemiology guidelines [23]. We searched the PubMed, EMBASE, Cochrane Library, and SCOPUS databases for studies in the English Language from inception to 9 May 2023, including conference abstracts. The reference lists of relevant review articles screened at the level of full texts were also manually searched. The literature search was conducted using the terms presented in Table S2. This meta-analysis was not registered previously to PROSPERO.

### 2.2. Study Selection

Using the Covidence platform [24], two independent reviewers (H.A.I and A.K., researchers with two years of experience) screened search results at the title and abstract levels using pre-defined criteria. The full texts of articles that met the initial abstract and title screening criteria were independently evaluated by the two reviewers using the predefined stepwise protocol (Table S2). In the case of disagreements, an independent arbiter, S.D., a musculoskeletal radiologist, provided supervision. Briefly, we included all texts that evaluated the prevalence of asymptomatic osteonecrosis of the hip in patients diagnosed with SLE and utilized MRI as the diagnostic modality. Studies were required to report either the prevalence of asymptomatic osteonecrosis in SLE patients directly or provide data that allowed for interpretation of the prevalence to be considered for inclusion in the subsequent analysis. Our initial search strategy involved the inclusion of keywords related to “MRI”, “SLE”, and “osteonecrosis” and their variations (Table S1). Animal studies, case reports, editorials, reviews, and studies reporting on symptomatic joints, non-hip joints, non-SLE populations, or non-MRI-based diagnoses were excluded (Table S2). Authors of studies that had data believed to be eligible but not discernible in the full text were contacted via email.

Qualifying MRI features include the following [25,26,27,28,29,30,31,32,33,34,35]: (1) a band or ring-shaped decreased signal intensity on T1 weighted images, (2) crescent-like high-intensity areas surrounding areas of low signal intensity on T2 weighted images, or (3) obvious femoral head epiphyseal deformities.

### 2.3. Data Extraction and Quality Assessment

Two reviewers (H.A.I and E.G, researchers with 2 years of experience) extracted data from eligible studies by using a standardized extraction form. Any disagreements were resolved by consensus. The data extracted include the year of study, the number of hips screened, the number of hips assessed as positive for asymptomatic osteonecrosis, the mean age of participants, no. of males, % of participants positive for antiphospholipid antibodies, % of participants reporting corticosteroid use, % of participants reporting pulse therapy, average corticosteroid dose, follow-up time, MRI protocol, definition of MRI-detected osteonecrosis, and MRI grading of osteonecrosis lesions.

Two reviewers (H.A.I and E.G) assessed the quality of studies using the STrengthening the Reporting of OBservational studies in Epidemiology (STROBE) tool (Table S6) [36]. The risk of bias was assessed using Hoy et al.’s tool for prevalence studies [37]. Each study was then classified as having a low (≥8 out of 10 score), moderate (6–7 out of 10 score), or high (≤5 out of 10 score) risk of bias (Table S7). Furthermore, we assessed the risk of bias using the Newcastle–Ottawa Quality Assessment Scale for observational studies (for studies designed as case controls or cohorts) [38], and the Cochrane RoB2 tool (for randomized control trials) [39], where applicable according to the original study design. Any disagreements were resolved by consensus, with enduring disagreements resolved by utilizing the lower rating.

### 2.4. Data Synthesis/Analysis

Meta-analyses were conducted to estimate the overall prevalence of MRI-detected asymptomatic osteonecrosis in hip joints of patients with SLE. A random effects model (restricted maximum likelihood estimator) using the Freeman–Tukey double arcsine transformed proportions as is suggested for proportional data [40] was implemented. Back-transformed prevalence values were then calculated after synthesis. The number needed to scan was calculated as the reciprocal of the prevalence values. Raw data have also been made available (Tables S4 and S5). 

We used forest plots (with back-transformed values), Q values, and the inconsistency index (I^2^ statistic) to estimate the between-study heterogeneity. We did not use threshold values for statistical heterogeneity determination due to the naturally high I^2^ levels in estimates of prevalence/proportional data [41]. Potential publication bias was assessed using Egger’s funnel plot asymmetry test and the trim-and-fill funnel plot methods. Potential influential/outlier studies were assessed using metrics including externally standardized residuals, Cook’s distances, and covariance ratios (Figure S1).

To explore sources of heterogeneity, subgroup analyses were conducted after (1) exclusion of influential/outlier studies, (2) after exclusion of studies exhibiting a high risk of bias. and based on (3) corticosteroid use, (4) the percentage of males, (5) follow-up time, (6) the level of expertise of image interpreters, and (7) the number of interpreters. Similarly, meta-regression analysis was conducted based on (1) daily corticosteroid dosage, (2) corticosteroid pulse therapy (where reported), (3) year of study, (4) follow-up time, and (5) proportion of individuals who have antiphospholipid antibody positivity (where reported). To enhance the outcomes of the classical meta-analysis, a Bayesian meta-analysis was performed. Similar to the conventional random effects model, this approach relies on the same foundational assumptions and also introduces a prior distribution that characterizes the uncertainty surrounding a specific effect measure. In cases of limited or insufficient available information, as for our meta-analysis, non-informative or weakly informative priors could be used. The meta-analysis likelihood summarizes both the data from included studies and the meta-analysis model (assuming random effects) [42,43,44]. 

A *p*-value less than 0.05 was considered significant. Analyses were conducted using R by H.A.I and E.G. (version 4.2.0; R Foundation for Statistical Computing; packages: metafor and meta version 3.4-0, and bayesmeta [45]).

## 3. Results

**Figure 1:**
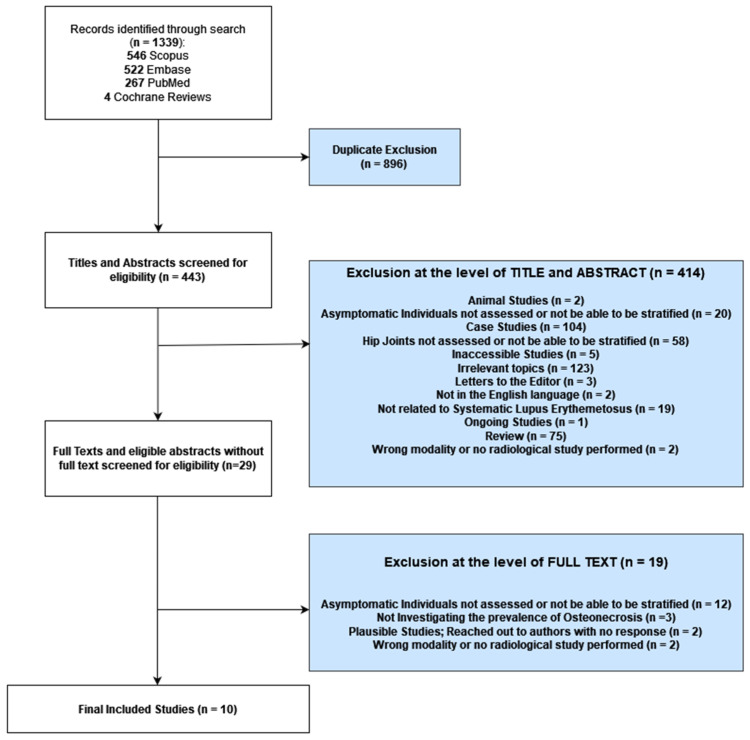
Flowchart for inclusion and exclusion of studies.

As shown in Figure 1, initially, our search yielded 1339 articles. Non-duplicate titles and abstracts (*n* = 443) were screened, with exclusions made for animal studies (*n* = 2), asymptomatic individuals not assessed or not able to be stratified (*n* = 20), case studies (*n* = 104), hip joints not assessed or not able to be stratified (*n* = 58), inaccessibility (*n* = 5), irrelevant topics (*n* = 123), letters to the editor (*n* = 3), not in the English language (*n* = 2), not related to SLE (*n* = 19), ongoing studies (*n* = 1), review articles (*n* = 75), and wrong modality/no radiological study performed (*n* = 2). Hence, 29 articles were selected for screening at the level of full texts. Eleven studies met our criteria for review [25,26,27,28,29,30,31,32,33,34,35]. The excluded articles and reasons for exclusions at the level of the full text are listed in Table S3.

### 3.1. Quality Assessment and Study Characteristics

**Figure 2:**
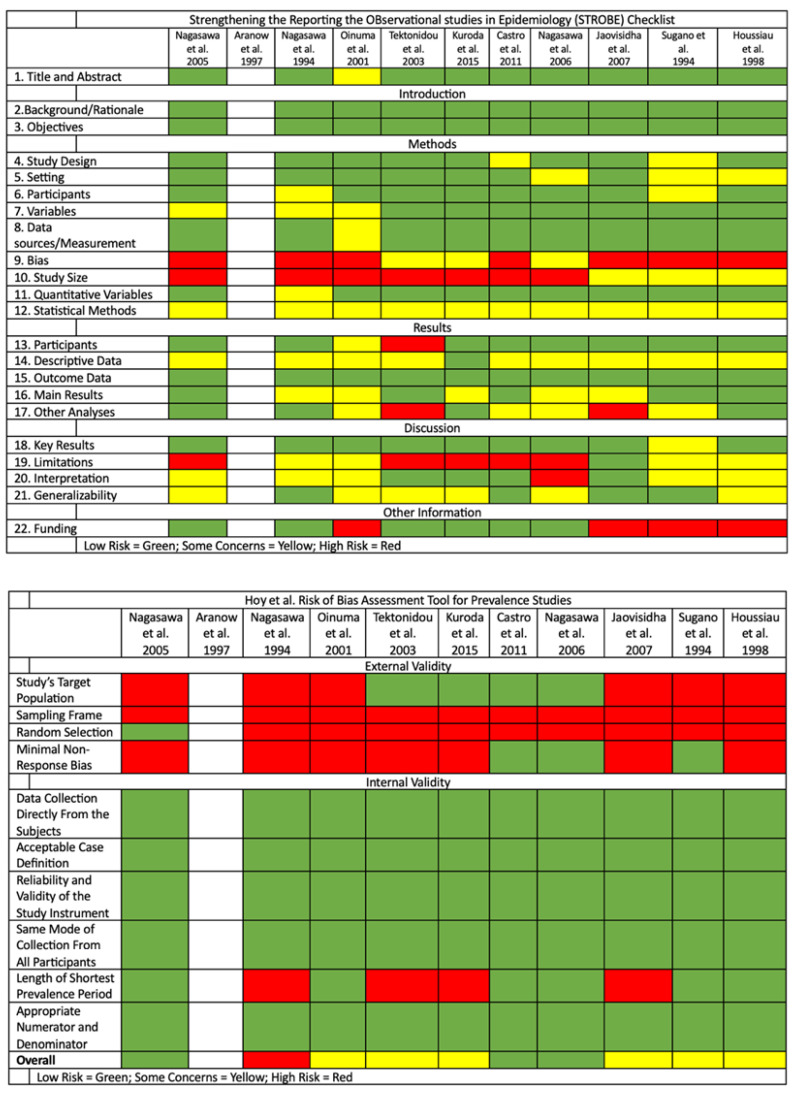
Quality and risk of bias assessment using the STROBE and Hoy et al. tools [25,26,27,28,29,30,31,32,33,34,35,36,37].

Details of the quality and risk of bias assessment are summarized in Figure 2 and Tables S8 and S9. Of the 11 studies included, we were unable to assess the risk of bias in 1 study due to unavailability of the full text, 1 study was judged to have a high risk of bias, and 6 studies were judged to have a moderate risk of bias. The remaining 3 studies were judged to have a low risk of bias.

The main characteristics of the included studies are presented in Table 1, Tables S3 and S4. One study exclusively included patients with no history of corticosteroid use, while nine studies encompassed patients who all had a history of corticosteroid use. Additionally, one study included both corticosteroid users and non-users which was divided into two distinct subgroups in our analyses. Sample sizes ranged from 22 to 156 hip joints (overall, 1006 hip joints), mean age ranged from 15 to 35 years, and the proportion of female participants ranged from 74% to 100%.

### 3.2. Meta-Analysis: Prevalence of Asymptomatic Hip Osteonecrosis

**Figure 3:**
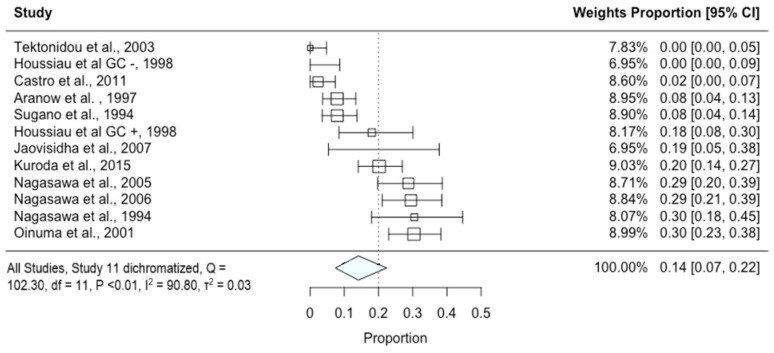
Forest plot of initial syntheses of all identified studies [25,26,27,28,29,30,31,32,33,34,35]. GC +: subset of participants receiving corticosteroid treatment; GC −: subset of participants not receiving corticosteroid treatment.

In our pooled data, the prevalence of asymptomatic osteonecrosis of the hip amongst patients with SLE was 14% (95% confidence interval (CI): 7%, 22%) (184 out of 1006 hip joints; number needed to scan (NNS): 7.1) (Figure 3). Restricted to analysis of studies with a low-to-moderate risk of bias (13%; 95% CI: 6%, 23%; 159 out of 708 hip joints; NNS: 7.7), a similar pooled estimate for the prevalence of asymptomatic osteonecrosis was found.

Furthermore, two studies [26,31] and the corticosteroid non-user subgroup of one study [32] were found to be influential/outlier studies according to several model diagnostic functions including the externally standardized residual and Cook’s distances (Figure S1). Restricted to analysis of studies excluding outliers, a pooled estimate of 21% (95% CI: 14%, 28%; NNS: 4.8) was found.

### 3.3. Publication Bias

**Figure 4:**
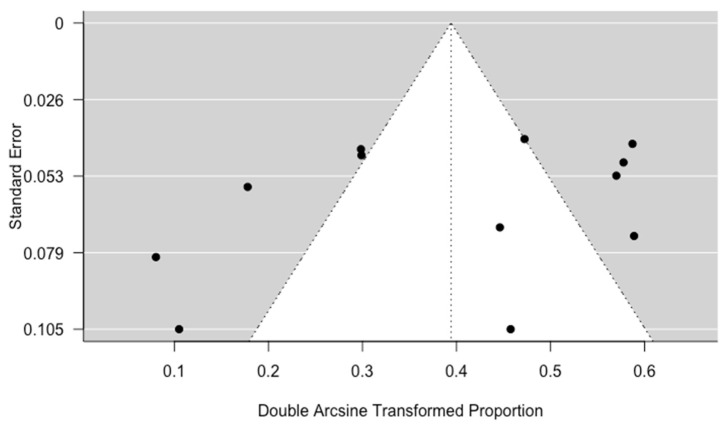
Funnel plot of initial syntheses of all identified studies; study 11 is dichotomized.

The Egger’s regression test for funnel plot asymmetry found no publication bias (*p*-value = 0.22) (Figure 4). The trim-and-fill method applied on a synthesis of all included studies suggested one missing study on the right side (Figure S2) and resulted in a pooled estimate of 16% (95% CI: 9%, 25%; NNS: 6.25).

### 3.4. Subgroup Analyses

The tests for interaction between subgroups (Table 2) showed no difference based on % of males (cutoff of 10%), follow-up time (12-month follow-up), musculoskeletal radiologist interpreter, the number of interpreters (1 vs. 2), the mean age of participants (30 and below vs above 30), and after exclusion of influential studies. However, there was a significantly higher prevalence of asymptomatic osteonecrosis in hip joints of patients with SLE with reported corticosteroid use than in patients with SLE without reported corticosteroid use (18% vs. 0%, *p*-value < 0.01).

### 3.5. Meta-Regression

**Figure 5:**
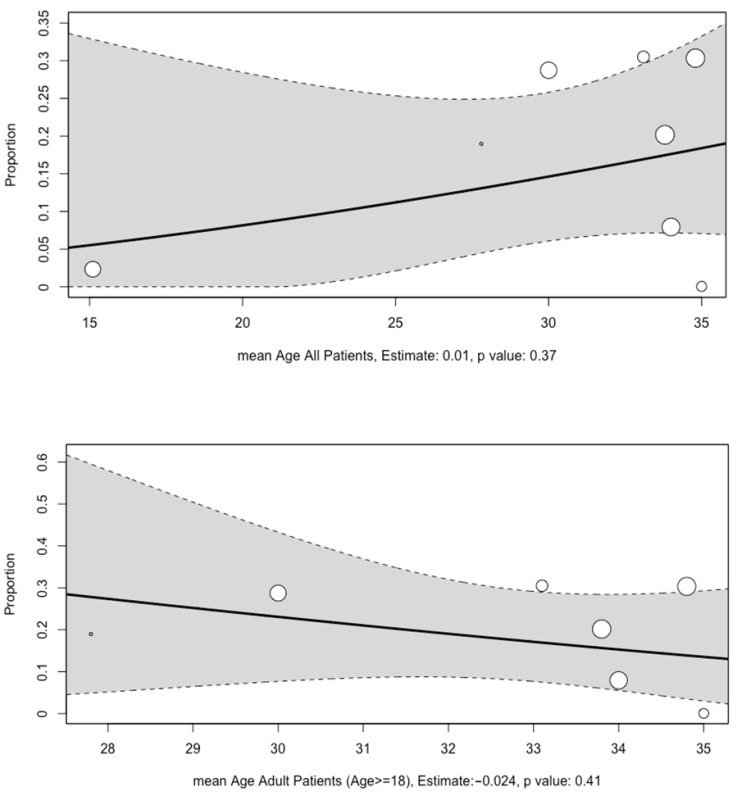
Meta-regression analysis conducted for the association between mean age of participants and age of adult participants and rate of asymptomatic osteonecrosis in hip joints of patients with SLE. The size of the circles represent the effect sizes; the solid line represents the line of best fit; the dotted lines represent the upper and lower limits of the 95% confidence interval.

**Figure 6:**
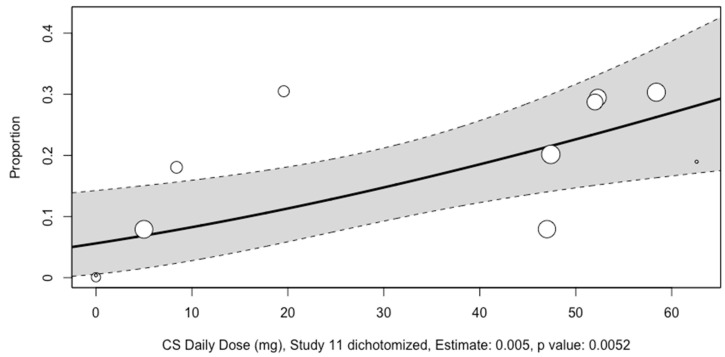
Meta-regression analysis conducted for the association between corticosteroid daily dose and rate of asymptomatic osteonecrosis in hip joints of patients with SLE. The size of the circles represent the effect sizes; the solid line represents the line of best fit; the dotted lines represent the upper and lower limits of the 95% confidence interval.

No associations were found between the mean age of participants, the mean age of adult participants, corticosteroid pulse therapy, the year of study, follow-up time, and prevalence of asymptomatic osteonecrosis (Figure 5 and Figures S3–S5). However, daily corticosteroid dose usage (10 studies, estimate per milligram of corticosteroid: 0.5%, *p*-value < 0.01) and the proportion of participants with antiphospholipid antibody positivity were found to be associated with the prevalence of asymptomatic osteonecrosis (Figure 6 and Figure S6).

Bayesian meta-analysis revealed that the mean odds of the quoted estimate was 0.39 (0.26; 0.52) (Figure S7). Thus, converting the odds obtained by Bayesian meta-analysis translated to an overall prevalence of 14%.

## 4. Discussion

This meta-analysis demonstrated that the prevalence of asymptomatic osteonecrosis of the hip in SLE as detected by MRI was 14%. Sensitivity analyses with the exclusion of influential studies and those with a high risk of bias were 21% and 13%, respectively. Subgroup analysis revealed that corticosteroid use may be associated with increased hip osteonecrosis prevalence in asymptomatic SLE patients. Additionally, meta-regression analyses revealed that dosage of corticosteroid use was positively associated with asymptomatic osteonecrosis. While our subgroup analysis found no osteonecrosis cases among SLE patients who are corticosteroid non-users, it is essential to recognize potential biases. Firstly, the non-corticosteroid group had only 60 hip joints. Secondly, these patients may have had milder SLE and insufficient follow-up. In the study including both corticosteroid users and non-users, follow-up durations ranged widely from 5 to 308 months [32]. It is plausible that the corticosteroid non-users may have had shorter follow-ups, potentially introducing bias. Furthermore, in another study involving only corticosteroid non-users, the follow-up period for patients was 6 months, which is among the shortest durations reported across all other studies. The meta-regression analysis revealed that there was no statistically significant difference in the prevalence of asymptomatic osteonecrosis based on patients’ age. This implies that the prevalence of asymptomatic osteonecrosis remains constant across different age groups and suggests that the burden of asymptomatic osteonecrosis on younger patients with the same prevalence might be greater due to potential greater long-term consequences.

SLE has been identified as the most frequently reported underlying disease linked to femoral head osteonecrosis among individuals who had previously received systemic steroids [5]. Given the relatively high prevalence of asymptomatic osteonecrosis in young patients with SLE, consideration must be given to strategies that can be employed to identify individuals with asymptomatic osteonecrosis of the hip in patients with SLE. Currently, there are no guidelines on SLE-associated asymptomatic osteonecrosis by the American College of Rheumatology and British Society of Rheumatology [46]. The European Alliance of Associations for Rheumatology [47,48], and Australian Rheumatology Associations, as well as independent systematic reviews of clinical practice guidelines [49], also do not address SLE-associated osteonecrosis. However, the Canadian Rheumatology Association has recommended no screening for patients who do not have clinical symptoms suggestive of osteonecrosis due to low-quality evidence regarding the progression of asymptomatic osteonecrosis to symptomatic osteonecrosis [50]. 

A study on the natural progression of asymptomatic osteonecrosis of the femoral head utilizing MRI, radiographs, and bone scans found that 55.9% of patients developed symptoms after an average of 2.27 years [11]. Additionally, larger osteonecrosis lesions had a higher likelihood of developing symptoms, emphasizing the importance of proactively identifying patients with asymptomatic osteonecrosis lesions to take less invasive measures before the onset of symptoms [11].

Though specific recommendations for screening are difficult to propose in light of our study due to the limited number and heterogeneity of the included studies, it is important to discuss future possibilities as the body of literature continues to grow. If deemed necessary, several modalities show potential for their use in osteonecrosis screening in individuals with SLE. For example, the use of radiographs may identify asymptomatic cases with multiple risk factors (corticosteroid use, high low-density lipoprotein, and antiphospholipid antibody positivity) [4]. Additionally, more sensitive modalities such as MRI can be used if combined with other cost-saving strategies to reduce the cost of imaging while still maintaining increased sensitivity. These may include focused imaging protocols specifically designed to screen for femoral head osteonecrosis [51] and single-time or low-frequency screening (e.g., after a pre-specified duration of disease or before corticosteroid initiation). 

To take advantage of any potential attempt to identify asymptomatic osteonecrosis in SLE patients, treatment of asymptomatic osteonecrosis may be pursued depending on lesion size [14,51] (those with ≥15% femoral head involvement) and location (those with laterally located lesions [11] or type C2 lesions as described by Sugano et al. [14]). Another study has suggested treatment for lesions spanning more than the medial two-thirds of the weight-bearing surface, exceeding the acetabular edge [52].

End-stage osteonecrosis requires total joint replacement. However, less invasive procedures can be considered if the lesion is detected early. These options include core decompression with bone marrow aspirate concentrate, bone grafting, and osteotomy [9]. Additionally, some studies suggest non-operative treatment (which may include the use of non-steroidal anti-inflammatory agents and partial weight-bearing) may be appropriate for asymptomatic individuals with less than 25% femoral head involvement, sparing the lateral two-thirds of the weight-bearing portion [53]. A study demonstrated that among patients who underwent total hip replacement, those with SLE [54] tended to be younger, had extended hospital stays, had an increased likelihood of complications, and presented a higher burden of comorbidities compared to their counterparts without SLE [54]. This study, along with our findings, emphasizes potential cost savings and efficient resource allocation through timely detection and intervention. 

Importantly, though SLE is the most common underlying autoimmune disease for osteonecrosis, future studies may also aim to find similar frequencies for other underlying diseases that increase osteonecrosis risk, such as coagulation disorders, sickle cell disease, and other connective tissue disorders [55]. Future studies may focus on modifiable risk factors that are specific to such diseases. Thus, larger scope data on the prevalence of osteonecrosis can prove helpful for secondary prevention and timely treatment for all-cause osteonecrosis with disease-specific considerations.

Our study has limitations. As only a single study included SLE patients without corticosteroid administration, no definitive conclusion can be drawn from a comparison between studies of SLE patients receiving corticosteroid therapy and the study of SLE patients not receiving corticosteroid therapy. Additionally, many of the included studies originate from a single geographical origin, which may further introduce biases to our estimation due to demographic and socioeconomical factors. The limited number of identified studies may have prevented an accurate estimate of asymptomatic hip joint osteonecrosis prevalence in SLE patients. In addition, as most of these studies were conducted 10+ years ago, the results of our study may not reflect the evolution of SLE epidemiology and MRI technology. One identified study lacked full-text availability, preventing a comprehensive assessment [25]. Moreover, the consistency and comprehensiveness of reports of corticosteroid treatment dosages differed among studies, complicating the interpretation of results, especially regarding the threshold dosage associated with osteonecrosis. Some of the studies had short follow-up periods, leading to an underestimation of prevalence. Furthermore, some studies such as Castro et al. [31] and Houssiau et al. [32] included patients with unilaterally eligible hips, thereby prohibiting a patient-level analysis, which may be important clinically as many individuals with unilateral osteonecrosis may have a propensity to develop osteonecrosis in the contralateral site, though individual joints are targeted in routine clinical practice. Lastly, assessing the cost-effectiveness of MRI scans, the transition from corticosteroid therapy to other available treatments, and suggesting optimal timing for performing MRI among asymptomatic patients based on existing data, presented challenges. As such, the authors do not make any specific recommendations based on the present meta-analysis.

In conclusion, our study has found that an overall asymptomatic osteonecrosis prevalence of 14% may be present in hip joints of SLE patients, with a possible higher prevalence in individuals treated with corticosteroids. Future studies may aim to further identify the prevalence and risk factors of the progression of asymptomatic disease to a symptomatic state and also the efficacy of potential early intervention. Additionally, further studies may choose to elaborate on the frequency of osteonecrosis in SLE patients for multiple sites (e.g., the knee and ankle joints) in order to provide a more holistic patient-level estimate, as there was a paucity of such data in our identified studies. As these are important considerations, the authors reserve from the proposal of any specific recommendations for the screening of individuals with SLE to prevent over-utilization of healthcare resources.

## Figures and Tables

**Table 1:** Main characteristics of included studies.

Number	Author	Year	Country	Osteonecrosis Positive	Total Screened Hips	Age (Mean)	% of Male Participants	Antiphospholipid Ab Positive	% of Participants Treated with Corticosteroid	% of Participants Treated with Pulse Therapy	Corticosteroid Dose (mg/day)	FUP Months
1	Nagasawa et al. [27]	2005	Japan	26	90	30	4	15.5	100	53.3	52	60
2	Aranow et al. [25]	1997	USA	11	132		0	NA	100	NA	5	12
3	Nagasawa et al. [28]	1994	Japan	14	46	33.1	17	NA	100	39.1	19.57	36
4	Oinuma et al. [29]	2001	Japan	44	144	34.8	5.8	NA	100	48.6	58.4	12
5	Tektonidou et al. [26]	2003	Greece	0	38	35	26	NA	NA	NA	0	6
6	Kuroda et al. [30]	2015	Japan	32	156	33.8	10.3	26.9	100	16.6	47.4	6
7	Castro et al. [31]	2011	Brazil	2	78	15.1	17.5	NA	100	95		24
8	Nagasawa et al. [33]	2006	Japan	32	108		8.3	NA		48.3	52.3	60
9	Jaovisidha et al. [34]	2007	Thailand	4	22	27.8	0	NA	100	18	62.6	1.3
10	Sugano et al. [35]	1994	Japan	10	120	24	3	NA	100	26	47	60
11	Houssiau et al. [32]	1998	Belgium	9	72	34	1	32.5	27.5	NA	6.01	100
11 CS +	Houssiau et al. [32]	1998	Belgium	9	50	NA	NA	NA	100	NA	8.4	100
11 CS −	Houssiau et al. [32]	1998	Belgium	0	22	NA	NA	NA	0	NA	0	100

NAs in the table indicate that the data were not extractable from the study.

**Table 2:** Sensitivity and subgroup analysis. Does corticosteroid use, sex, follow-up time, number of interpreters, and interpreters including MSK radiologists affect the detection rate of asymptomatic avascular osteonecrosis of the hip?

	Sensitivity Analysis (Number of Studies ^a^)	Number of Hips	Proportion (95% CI)	T^2^	I^2^	Heterogeneity *p* Value
All Studies	12	1006	0.14 (0.07, 0.22)	0.03	90.8%	Ref
Excluding influential studies	9	868	0.21 (0.14, 0.28)	0.01	82%	0.228
Excluding studies with unknown/high risk of bias	10	828	0.13 (0.06, 0.23)	0.03	91.4%	0.910
	Subgroup analysis (Number of studies)	Number of hips	Proportion (95% CI)	T^2^	I^2^	*p* value
Corticosteroid use						
Use reported	10	946	0.18 (0.11, 0.26)	0.02	87.5%	**<0.01**
Use not reported	2	60	0.00 (0.00, 0.04)	-	-	
% Male participants ^b^						
>10%	4	318	0.10 (0.00, 0.28)	0.05	93.6%	0.316
<10%	6	688	0.20 (0.11, 0.30)	0.02	87.7%	
Follow-up time						
>12 months	7	514	0.15(0.06, 0.27)	0.04	90.6%	0.877
<12 months	5	492	0.13 (0.04, 0.27)	0.03	92.4%	
MSK radiologist interpreter						
Yes	2	100	0.08 (0.00, 0.29)	0.03	81.7%	0.612
No	5	420	0.14 (0.04, 0.28)	0.04	91.9%	
Number of interpreters						
One	3	228	0.12(0.10, 0.25)	0.03	87.1%	0.831
Two	4	184	0.09 (0.00, 0.28)	0.05	89.8%	
Age						
30 and below	3	190	0.14 (0.02, 0.35)	0.04	90.0%	0.947
Above 30	5	576	0.15 (0.04, 0.31)	0.04	93.9%	

^a^ Houssiau et al.’s study [32] is counted twice as two subgroups of glucocorticoid users and non-users. ^b^ For % of male participants subgroup analysis, Houssiau et al.’s study was not dichotomized due to unavailability of the data.

## Data Availability

The data extracted from included studies, which were subsequently used for analysis, are available as part of the Supplementary Material.

## Supplementary Materials

The following supporting information can be downloaded at: https://www.mdpi.com/article/10.3390/diagnostics14030279/s1, Table S1: Search Terms for the PubMed, Embase, Cochrane, and Scopus databases. Table S2: Study selection, Inclusion and Exclusion Criteria, and the reasons for exclusion of Studies at the full text Level. Table S3: Reasons for Exclusion of Papers at the full-text Level [56,57,58,59,60,61,62,63,64,65,66,67,68,69,70,71,72,73]. Table S4: Data Extraction from Eligible Studies. Table S5: MRI Protocol and Osteonecrosis Imaging Definitions Utilized by each Study. Table S6: STROBE Checklist used for Quality Assessment. Table S7: Hoy et al.’s Risk of Bias Assessment Tool for Prevalence Studies. Table S8: Quality Assessment based on the Newcastle-Ottawa Scale of Studies. Table S9: Cochrane Risk of Bias Assessment. Figure S1: Plots showing influence measures of the included studies. Figure S2: Trim and Fill Method for Meta-analysis conducted on all identified studies. Figure S3: Meta-regression analysis conducted based on percent of participants received corticosteroid (CS) pulse therapy. Figure S4: Meta-regression analyses conducted based on year of study. Figure S5: Meta-regression analyses conducted based on follow-up time. Figure S6: Meta-regression analyses conducted based on percent of participants with positive APL Antibody. Figure S7: Bayesian Meta-analysis forest plot, All Participants. File S1: PRISMA Checklist.

## Abbreviations

SLE: systemic lupus erythematosus; MRI: magnetic resonance imaging.
