# Supplementary material for: Screening for Asymptomatic Osteonecrosis of the Hip in Systemic Lupus Erythematous: A Systematic Review and Meta-Analysis of MRI-Based Prevalence
Source: Diagnostics (Basel). 2024 Jan 27;14(3):279. doi: 10.3390/diagnostics14030279 (PMC10855524; doi:10.3390/diagnostics14030279)
Supplement: Supplementary file 1 [file diagnostics-14-00279-s001.zip › Supplemental Materials.pdf]

Supplementary Tables

Table S1. Search Terms for the PubMed, Embase, Cochrane, and Scopus databases

**PubMed**	("Magnetic Resonance Imaging"[mh:noexp] OR "diffusion magnetic resonance imaging"[mh] OR "echo-planar imaging"[mh] OR "Magnetic resonance imaging"[tw] OR "MRI"[tw] OR "nuclear magnetic resonance"[tw] OR "magnetic resonance image*"[tw] OR "echo-planar imaging"[tw] OR "echo-planar image*"[tw] OR "MR tomography"[tw])<br>AND<br>("Femur Head Necrosis"[Mesh] OR "osteonecrosis"[mh:noexp] OR "osteonecrosis"[tw] OR "osteonecrotic"[tw] OR (("necrosis"[tw] OR "necrotic"[tw]) AND ("femur head"[mh] OR "femur"[tw] OR "femoral"[tw] OR "hip"[tw] OR "hips"[tw] OR "hip joint"[mh] OR "acetabulofemoral joint"[tw])))<br>AND

	("Lupus Erythematosus, Systemic"[mh:noexp] OR "lupus"[tw] OR "SLE"[tw] OR "Libman sacks"[tw])<br><br>AND eng[la]
**Embase**	('nuclear magnetic resonance imaging'/de OR 'diffusion weighted imaging'/exp OR 'echo planar imaging'/exp OR 'Magnetic resonance imaging':ti,ab OR 'MRI':ti,ab OR 'nuclear magnetic resonance':ti,ab OR 'magnetic resonance image*':ti,ab OR 'echo-planar imaging':ti,ab OR 'echo-planar image*':ti,ab OR 'MR tomography':ti,ab)<br><br>AND<br><br>('femur head necrosis'/exp OR 'bone necrosis'/de OR 'osteonecrosis':ti,ab OR 'osteonecrotic':ti,ab OR (('necrosis':ti,ab OR 'necrotic':ti,ab) AND ('femoral head'/de OR 'femur':ti,ab OR 'femoral':ti,ab OR 'hip':ti,ab OR 'hips':ti,ab OR 'hip'/de OR 'acetabulofemoral joint':ti,ab)))<br><br>AND<br><br>('systemic lupus erythematosus'/de OR 'lupus':ti,ab OR 'SLE':ti,ab OR 'Libman sacks':ti,ab)

	AND [english]/lim
**Cochrane**	([mh ^"Magnetic Resonance Imaging"] OR [mh "diffusion magnetic resonance imaging"] OR [mh "echo-planar imaging"] OR "Magnetic resonance imaging":ti,ab,kw OR "MRI":ti,ab,kw OR "nuclear magnetic resonance":ti,ab,kw OR "magnetic resonance image*":ti,ab,kw OR "echo-planar imaging":ti,ab,kw OR "echo-planar image*":ti,ab,kw OR "MR tomography":ti,ab,kw)<br><br>AND<br><br>([mh "Femur Head Necrosis"] OR [mh ^"osteonecrosis"] OR "osteonecrosis":ti,ab,kw OR "osteonecrotic":ti,ab,kw OR (("necrosis":ti,ab,kw OR "necrotic":ti,ab,kw) AND ([mh "femur head"] OR "femur":ti,ab,kw OR "femoral":ti,ab,kw OR "hip":ti,ab,kw OR "hips":ti,ab,kw OR [mh "hip joint"] OR "acetabulofemoral joint":ti,ab,kw)))<br><br>AND

	([mh ^"Lupus Erythematosus, Systemic"] OR "lupus":ti,ab,kw OR "SLE":ti,ab,kw OR "Libman sacks":ti,ab,kw)
**Scopus**	TITLE-ABS-KEY({Magnetic resonance imaging} OR {MRI} OR {nuclear magnetic resonance} OR "magnetic resonance image*" OR {echo-planar imaging} OR "echo-planar image*" OR {MR tomography})<br><br>AND<br><br>TITLE-ABS-KEY({osteonecrosis} OR {osteonecrotic} OR (({necrosis} OR {necrotic}) AND ({femur} OR {femoral} OR {hip} OR {hips} OR {acetabulofemoral joint})))<br><br>AND<br><br>TITLE-ABS-KEY({lupus} OR {SLE} OR {Libman sacks})<br><br>AND LANGUAGE(english)

Table S2. Study selection, Inclusion and Exclusion Criteria, and the reasons for exclusion of Studies at the full text Level

1.Is this study a case report, case series, review, editorial or animal study?<br>No -> proceed to 2<br>Yes -> exclude
2.is this study in English language?<br>No -> exclude<br>Yes -> proceed to 3
3.Does the study enrolls patients with diagnosis of SLE?<br>No -> exclude<br>Yes -> proceed to 4
4.Does the study utilize MRI for the diagnosis of osteonecrosis of hip joint?<br>No -> exclude<br>Yes -> proceed to 5
5.Does the study provide data on the prevalence of MRI-diagnosed asymptomatic osteonecrosis in patients with SLE?<br>No -> exclude<br>Yes -> proceed to data extraction

Table S3. Reasons for Exclusion of Papers at the full-text Level [56-73].

Author (Date)	DOI (or other identifier if absent)	Reason For Exclusion (As assessed by two reviewers)
Jeong et al (2017) [56]	10.1111/1756-185X.13065	Asymptomatic Individuals could not be stratified. Additionally, the study looked at time to multifocal osteonecrosis , which may underestimate osteonecrosis prevalence by increasing threshold to be considered a 'case'
Bogmat et al (2018) [57]	10.1186/s12969-018-0265-6	Cannot determine if the positive case was asymptomatic. Additionally, it could not be determined the modality used to diagnose osteonecrosis .
Klippel et al (1979) [58]	10.1016/0002-9343(79)90077-9	MRI was not performed for these study individuals. However, stratification by hip joint and symptomatology is possible
Sekiya et al (2010) [59]	10.1007/s00296-009-1194-y	Asymptomatic individuals were excluded from the initial cohort. No figure is given as to how many total participants were screened beforehand as well.
Sakamoto et al (1997) [60]	10.1302/0301-620X.79B2.7179	Asymptomatic Individuals could not be stratified.
Fialho et al (2007) [61]	10.1177/0961203307076771	Asymptomatic Individuals could not be stratified. Symptomatology was determined on a per-participant and not a per-hip basis.
Shigemura et al (2011) [62]	10.1093/rheumatology/ker277	Asymptomatic Individuals could not be stratified from the symptomatic individuals. Multiple anatomical positions were evaluated, though hip joints were reported separately (but not further subdivided into symptomatic/asymptomatic)
Hurley et al (1974) [63]	NO DOI, PubMed ID: 4424118	Individuals were symptomatic at the time of diagnosis. MRI was not used as the modality of choice for diagnosis.
Chinnadurai et al (2017) [64]	10.4103/jfmpc.jfmpc_1234_19	All participants described were symptomatic. Computed Tomography was used in addition to MRI
Bergstein et al (1974) [65]	10.1016/s0022-3476(74)80281-7	MRI was not used as the modality of choice for diagnosis. Multiple site involvement was also investigated and stratification for asymptomatic hip joints was not feasible.
Hagiwara et al (2015) [66]	10.1002/jmri.24953	Individuals with priorly diagnosed disease as well as volunteer participants were recruited. As such, this study does not investigate prevalence but to investigate the role of corticosteroids on patients with and without osteonecrosis

Lee et al (2014) [67]	10.1177/0961203313512880	osteonecrosis was diagnosed using clinical symptoms as well as radiographic evidence. Asymptomatic osteonecrosis individuals were not explicity identified
Nawata et al (2018) [68]	10.1093/rheumatology/key009	Though the terminology screening is used, it is not explicitly stated that individuals in this study were asymptomatic at the time of examination
Nakamura et al (2010) [69]	10.1002/art.27236	Though the terminology screening is used, it is not explicitly stated that individuals in this study were asymptomatic at the time of examination
Yamamoto et al (2011) [70]	10.1002/jmri.22685	The aim of the study was not to establish prevalence. Asymptomatic Participants were compared to healthy volunteers, with no initial figures on screening.
Oh et al (2004) [71]	10.1016/S0899-7071(03)00192-X	Participants with Osteonecrosis were recruited from the onset. As such, prevalence was not established. Those who were recruited, were screened based on pathological and clinical criteria; symptomatology was not described.
Nakamura et al (2010) [72]	10.1177/0961203310372951	Study feasibly has data that can be stratified. Author was contacted (5/25/2023)
Chen et al (2010) [73]	10.1177/1759720X211002677	Study feasibly has data that can be stratified. Author was contacted (5/25/2023)

Table S4. Data Extraction from Eligible Studies

Number	Author	Year	Osteonecrosis Positive (n)	Screened (n)	Age	Male	APLAb Positive Participants %	CS use %	Pulse Therapy %	CS Dose (mg/day)	FUP months	FUP and CS dosage and other study details	Radiography Data	MRI grading of osteonecrosis lesions
1	Nagasawa et al.	2005	26	90	30	4	15.5	100	53.3	52	60	FUP after initiation of high dose CS, mean initial dose (mg/day) reported	All patients underwent radiographs, asymptomatic osteonecrosis was defined as absence of pain and or **radiographic abnormalities**, none of the MRI positive osteonecrosis patients had radiographic features of osteonecrosis	No MRI grading available
2	Aranow et al.	1997	11	132		0	NA	100		5	12	first MRI=>6months after initiation of CS, FUP for 12 months, min CS dose used for >= 6months reported(mg/day)	Radiographs were done only for those with MRI positive osteonecrosis	One lesion was MRI class B, the remaining lesions were class A.
3	Nagasawa et al.	1994	14	46	33.1	17	NA	100	39.1	19.57	36	Unknown FUP details, average daily dose of CS reported (md/day)	All patients underwent radiographs, asymptomatic osteonecrosis were defined as absence of pain and or **abnormal radiographs**, none of the MRI positive asymptomatic	No MRI grading available

													patients had abnormal radiographs	
4	Oinuma et al.	2001	44	144	34.8	5.8	NA	100	48.6	58.4	12	FUP after initiation of high dose CS, Initial mean CS dose reported(mg/day)	No radiographs	No MRI grading available
5	Tektonidou et al.	2003	0	38	35	26	NA	NA		0	6	Unknown FUP details, none of the patients had history of CS use	Radiographs done in patients that osteonecrosis was demonstrated by MRI	NA
6	Kuroda et al.	2015	32	156	33.8	10.3	26.9	100	16.6	47.4	6	FUP after initiation of high dose CS for newly diagnosed cases, mean initial dose of CS reported (mg/day)	All patients underwent radiography, asymptomatic osteonecrosis was defined as absence of pain and or abnormal radiographs, none of the MRI positive asymptomatic patients had abnormal radiographs	No MRI grading available
7	Castro et al.	2011	2	79	15.1	17.5	NA	100	95	NA	24	max daily CS dose reported: 1.1/kg, Juvenile SLE, one patient with previous diagnosis of osteonecrosis included	No radiographs	No concrete MRI grading available for all patients
8	Nagasawa et al.	2006	32	108		8.3	NA		48.3	52.3	60	FUP after initiation of high dose CS for newly diagnosed SLE, initial daily dose of CS reported (mg/day), Warfarin treatment in a	asymptomatic patients were defined as those without pain and or abnormal radiographs, so none of the MRI	No MRI grading available

												subgroup of patients,	positive asymptomatic patients had abnormal radiographs	
9	Jaovisidha et al.	2007	4	22	27.8	0	NA	100	18	62.6	1.3	FUP after initiation of CS for newly diagnosed cases, median of average daily CS dose reported (mg/day)	All patients underwent radiography and MRI	Three of four hips had osteonecrosis grade 1, and one had grade 2
10	Sugano et al.	1994	10	120	34	3	NA	100	26	47	60	The period from diagnosis of SLE to detection of osteonecrosis lesion: 9 months to 5 years, Study FUP for 3 to 7 years, mean of maximum daily CS dose excluding pulse therapy,	All patients underwent radiography and MRI, none of them had abnormal radiographic finding	According to ARCO international classification 6 hips with grade A, 2 hips with grade B, and 2 hips with grade C
11	Houssiau et al	1998	9	72	34	1	32.5	27.5	NA	6.01	100	Unknown FUP details, mean daily CS dose reported (mg/day)	No radiographs	No MRI grading available
11 CS +	Houssiau et al	1998	9	50	NA	NA	NA	100	NA	8.4	100	--	--	
11 CS -	Houssiau et al	1998	0	22	NA	NA	NA	0	NA	0	100	--	--	
	Abbreviations: CS= Corticosteroid, FUP= Follow up, APLAb: Anti phospholipid antibody													

Table S5. MRI Protocol and Osteonecrosis Imaging Definitions Utilized by each Study

Study Number	Author	MRI protocol	Osteonecrosis definition
1	Nagasawa et al.	T1 axial and coronal	band or ring-formed decreased signal intensity area
2	Aranow et al.		
3	Nagasawa et al.	1.5 Tesla, thickness 20 mm, T1 axial and coronal, echo time 20 ms, repetition time 400-600 ms	A band or ring-formed decreased signal area surrounding an area of high signal or a homogenous or unhomogenous area of low signal intensity
4	Oinuma et al.	0.5 Tesla, T1 , spin echo, STIR, "T1:echo time 18-40 ms, repetition time 300-400 ms" , "STIR: echo time 30-42 ms, repetition time 1500-3000 ms"	well demarcated, band- like zones of decreased signal intensity on T1, spin echo images and band-like zones of increased signal intensity on STIR
5	Tektonidou et al.	1.5 Tesla, T1, T2/proton density weighted, STIR,	Early osteonecrosis: the presence of a low-intensity band or rim in the subchondral zone of the femoral head (band sign), intermediate osteonecrosis: presence of a geographic area of decreased signal intensity with slightly diffuse margins (with or without a double-line sign), advanced osteonecrosis combination of the above findings with collapse and joint congruity
6	Kuroda et al.	T1	band-like low-signal area was seen in the femoral head on MRI T1-weighted images
7	Castro et al.	1.5 Tesla, T1, T2 with fat suppression, STIR. Coronal T1 repetition times 550–650 ms and echo times 10–12 ms. Coronal T2 with fat suppression repetition time 1900–2100 ms and echo times of 40– 60 ms. Sagittal T1 repetition times of 550–650 ms and echo times of 10–12 ms. Section thickness 4 mm. Whole-body STIR repetition time 4900–5000 ms and an echo times of 60–70 ms. Section thickness 6 mm	band or ring-formed decreased signal area surrounding an area of high signal in T1 images. In epiphyseal locations, low signal intensity and epiphyseal deformities: more advanced ischemic marrow lesions. T2 weighted fat-suppressed images: crescentic areas of high signal intensity surrounding an area of low signal intensity in the weight-bearing portion of the femoral head, or collapse of the femoral head
8	Nagasawa et al.	T1 spin echo pulse sequence	a band or ring-formed decreased signal intensity area

9	Jaovisidha et al.	1.5 Tesla, "coronal T1 spin echo, echo time 9-14, repetition time: 540" , "T2 fat supression echo time 81.4-89, repetition time: 3600-3900", section thickness 3, "sagittal T1 spin echo, echo time 9-14, repetition time: 440-582", section thickness 3	focal area of homogeneous/inhomogeneous band like low signal intensity on T1 or detectable “double line sign” change on T2
10	Sugano et al.	0.5 Tesla, T1 repetition time 600 ms, echo time 20 ms, and T2 repetition time 1500 ms, echo time 100 ms, section thickness 5,	Low intensity band demarcating the normal fat intensity area in femoral head on T1
11	Houssiau et al	0.5 T, T1 spin echo coronal, 5 mm thickness, echo time 20 ms, repetition time 265 ms	an area of high or partially high signal intensity circumscribed by a low-signal-intensity rim on T1, In epiphyseal locations: lesions with low signal intensity and frank epiphyseal deformities: more advanced ischemic marrow lesions

Abbreviations: ms= milliseconds

Table S6. STROBE Checklist used for Quality Assessment

	Item No.	Recommendation
**Title and abstract**	1	(*a*) Indicate the study's design with a commonly used term in the title or the abstract
		(*b*) Provide in the abstract an informative and balanced summary of what was done and what was found
**Introduction**		
Background/rationale	2	Explain the scientific background and rationale for the investigation being reported
Objectives	3	State specific objectives, including any prespecified hypotheses
**Methods**		
Study design	4	Present key elements of study design early in the paper
Setting	5	Describe the setting, locations, and relevant dates, including periods of recruitment, exposure, follow-up, and data collection
Participants	6	(*a*) *Cohort study*—Give the eligibility criteria, and the sources and methods of selection of participants. Describe methods of follow-up<br>*Case-control study*—Give the eligibility criteria, and the sources and methods of case ascertainment and control selection. Give the rationale for the choice of cases and controls.<br>*Cross-sectional study*—Give the eligibility criteria, and the sources and methods of selection of participants
		(*b*) *Cohort study*—For matched studies, give matching criteria and number of exposed and unexposed<br>*Case-control study*—For matched studies, give matching criteria and the number of controls per case
Variables	7	Clearly define all outcomes, exposures, predictors, potential confounders, and effect modifiers. Give diagnostic criteria, if applicable
Data sources/ measurement	8*	For each variable of interest, give sources of data and details of methods of assessment (measurement). Describe comparability of assessment methods if there is more than one group
Bias	9	Describe any efforts to address potential sources of bias
Study size	10	Explain how the study size was arrived at
*Give information separately for exposed and unexposed groups.		

Note: An Explanation and Elaboration article discusses each checklist item and gives methodological background and published examples of transparent reporting. The STROBE checklist is best used in conjunction with this article (freely available on the Web sites of PLoS Medicine at http://www.plosmedicine.org/, Annals of Internal Medicine at http://www.annals.org/, and Epidemiology at http://www.epidem.com/). Information on the STROBE Initiative is available at www.strobe-statement.org.

Table S7. Hoy et al's Risk of Bias Assessment Tool for Prevalence Studies. This tool is designed to assess the risk of bias in population-based prevalence studies. Please read the additional notes for each item when initially using the tool. Note: If there is insufficient information in the article to permit a judgement for a particular item, please answer No (HIGH RISK) for that particular item.

Risk of bias item	Criteria for answers (please circle one option)	Additional notes and examples
External Validity		
1. Was the study's target population a close representation of the national population in relation to relevant variables, e.g., age, sex, occupation?	• Yes (LOW RISK): The study's target population was a close representation of the national population.<br>• No (HIGH RISK): The study's target population was clearly NOT representative of the national population.	The target population refers to the group of people or entities to which the results of the study will be generalised. Examples:<br>• The study was a national health survey of people 15 years and over and the sample was drawn from a list that included all individuals in the population aged 15 years and over. The answer is: Yes (LOW RISK).<br>• The study was conducted in one province only, and it is not clear if this was representative of the national population. The answer is: No (HIGH RISK).<br>• The study was undertaken in one village only and it is clear this was not representative of the national population. The answer is: No (HIGH RISK).
2. Was the sampling frame a true or close representation of the target population?	• Yes (LOW RISK): The sampling frame was a true or close representation of the target population.<br>• No (HIGH RISK): The sampling frame was NOT a true or close representation of the target population.	The sampling frame is a list of the sampling units in the target population and the study sample is drawn from this list. Examples:<br>• The sampling frame was a list of almost every individual within the target population. The answer is: Yes (LOW RISK).<br>• The cluster sampling method was used and the sample of clusters/villages was drawn from a list of all villages in the target population. The answer is: Yes (LOW RISK).<br>• The sampling frame was a list of just one particular ethnic group within the overall target population, which comprised many groups. The answer is: No (HIGH RISK).
3. Was some form of random selection used to select the sample, OR was a census undertaken?	•Yes (LOW RISK): A census was undertaken, OR some form of random selection was used to select the sample (e.g. simple random sampling, stratified random sampling, cluster sampling, systematic sampling).<br>• No (HIGH RISK): A census was NOT undertaken, AND some form of random selection was NOT used to select the sample	A census collects information from every unit in the sampling frame. In a survey, only part of the sampling frame is sampled. In these instances, random selection of the sample helps minimise study bias. Examples:<br>• The sample was selected using simple random sampling. The answer is: Yes (LOW RISK).<br>• The target population was the village and every person in the village was sampled. The answer is: Yes (LOW RISK).<br>• The nearest villages to the capital city were selected in order to save on the cost of fuel. The answer is: No (HIGH RISK).
4. Was the likelihood of non-response bias minimal?	• Yes (LOW RISK): The response rate for the study was >/=75%, OR, an analysis was performed that showed no significant difference in relevant demographic characteristics	Examples:<br>• The response rate was 68%; however, the researchers did an analysis and found no significant difference between responders and non-responders in terms of age, sex,

	between responders and non-responders<br>• No (HIGH RISK): The response rate was <75%, and if any analysis comparing responders and non-responders was done, it showed a significant difference in relevant demographic characteristics between responders and non-responders	occupation and socio-economic status. The answer is: Yes (LOW RISK).<br>• The response rate was 65% and the researchers did NOT carry out an analysis to compare relevant demographic characteristics between responders and non-responders. The answer is: No (HIGH RISK).<br>• The response rate was 69% and the researchers did an analysis and found a significant difference in age, sex and socio-economic status between responders and non-responders. The answer is: No (HIGH RISK).
Internal Validity		
5. Were data collected directly from the subjects (as opposed to a proxy)?	• Yes (LOW RISK): All data were collected directly from the subjects.<br>• No (HIGH RISK): In some instances, data were collected from a proxy.	A proxy is a representative of the subject. Examples:<br>• All eligible subjects in the household were interviewed separately. The answer is: Yes (LOW RISK).<br>• A representative of the household was interviewed and questioned about the presence of low back pain in each household member. The answer is: No (HIGH RISK).
6. Was an acceptable case definition used in the study?	• Yes (LOW RISK): An acceptable case definition was used.<br>• No (HIGH RISK): An acceptable case definition was NOT used.	• For a study on low back pain, the following case definition was used: "Low back pain is defined as activity-limiting pain lasting more than one day in the area on the posterior aspect of the body from the bottom of the 12th rib to the lower gluteal folds." The answer is: Yes (LOW RISK).<br>• For a study on back pain, there was no description of the specific anatomical location „back" referred to. The answer is: No (HIGH RISK).<br>• For a study on osteoarthritis, the following case definition was used: "Symptomatic osteoarthritis of the hip or knee, radiologically confirmed as Kellgren-Lawrence grade 2-4". The answer is: LOW RISK.
7. Was the study instrument that measured the parameter of interest (e.g., prevalence of low back pain) shown to have reliability and validity (if necessary)?	• Yes (LOW RISK): The study instrument had been shown to have reliability and validity (if this was necessary), e.g., test-re-test, piloting, validation in a previous study, etc.<br>• No (HIGH RISK): The study instrument had NOT been shown to have reliability or validity (if this was necessary).	• The authors used the COPCORD questionnaire, which had previously been validated. They also tested the inter-rater reliability of the questionnaire. The answer is: Yes (LOW RISK).<br>• The authors developed their questionnaire and did not test this for validity or reliability. The answer is: No (HIGH RISK).
8. Was the same mode of data collection used for all subjects?	• Yes (LOW RISK): The same mode of data collection was used for all subjects.<br>• No (HIGH RISK): The same mode of data collection was NOT used for all subjects.	The mode of data collection is the method used for collecting information from the subjects. The most common modes are face-to-face interviews, telephone interviews, and self-administered questionnaires. Examples:<br>• All eligible subjects had a face-to-face interview. The answer is: Yes (LOW RISK).<br>• Some subjects were interviewed over the telephone and some filled in postal questionnaires. The answer is: No (HIGH RISK).
9. Was the length of the shortest prevalence period for the parameter of interest appropriate?	• Yes (LOW RISK): The shortest prevalence period for the parameter of interest was appropriate (e.g., point prevalence, one-week prevalence, one-year prevalence).<br>• No (HIGH RISK): The shortest prevalence period for the	The prevalence period is the period that the subject is asked about e.g. "Have you experienced low back pain over the previous year?" In this example, the prevalence period is one year. The longer the prevalence period, the greater the likelihood of the subject forgetting if they experienced the symptom of interest (e.g., low back pain). Examples:<br>• Subjects were asked about pain over the past week. The answer is: Yes (LOW RISK).

	parameter of interest was not appropriate (e.g., lifetime prevalence)	• Subjects were only asked about pain over the past three years. The answer is: No (HIGH RISK).
10. Were the numerator(s) and denominator(s) for the parameter of interest appropriate?	• Yes (LOW RISK): The paper presented appropriate numerator(s) AND denominator(s) for the parameter of interest (e.g., the prevalence of low back pain).<br>• No (HIGH RISK): The paper did present numerator(s) AND denominator(s) for the parameter of interest but one or more of these were inappropriate.	There may be errors in the calculation and/or reporting of the numerator and/or denominator. Examples:<br>• There were no errors in the reporting of the numerator(s) AND denominator(s) for the prevalence of low back pain. The answer is: Yes (LOW RISK).<br>• In reporting the overall prevalence of low back pain (in both men and women), the authors accidentally used the population of women as the denominator rather than the combined population. The answer is: No (HIGH RISK).
11. Summary item on the overall risk of study bias		
• LOW RISK OF BIAS: Further research is very unlikely to change our confidence in the estimate.<br>• MODERATE RISK OF BIAS: Further research is likely to have an important impact on our confidence in the estimate and may change the estimate.<br>• HIGH RISK OF BIAS: Further research is very likely to have an important impact on our confidence in the estimate and is likely to change the estimate.		

Table S8. Quality Assessment based on the Newcastle-Ottawa Scale of Studies

Quality assessment based on the Newcastle-Ottawa Scale of studies											
	1.Nagasawa et al.2005	2.Aranow et al. 1997	3.Nagasawa et al.1994	4.Oinuma et al.2001	5.Oinuma et al.	6.Kuroda et al.2015	7.Castro et al.2011	8.Nagasawa et al.2006	9.Jaovisidha et al.2007	10. Sugano et al.	11.Houssiau et al.1998
Selection	****		***	**	***	**	***			**	***
Comparability	*		-	*	*	*	*			*	*
Outcome/Exposure	**		***	***	***	***	***			***	***
Total Quality Scores	7		6	6	7	6	7			6	7

Table S9. Cochrane Risk of Bias Assessment

Cochrane risk-of-bias tool for randomized trials version 2	
	Nagasawa et al.2006
Randomization process	!
Deviation from intended interventions	!
Missing outcome data	+
Measurement of the outcome	+
Selection of the reported result	!
overall	!

Color	Risk
+	Low Risk
!	Some Concerns
-	High Risk

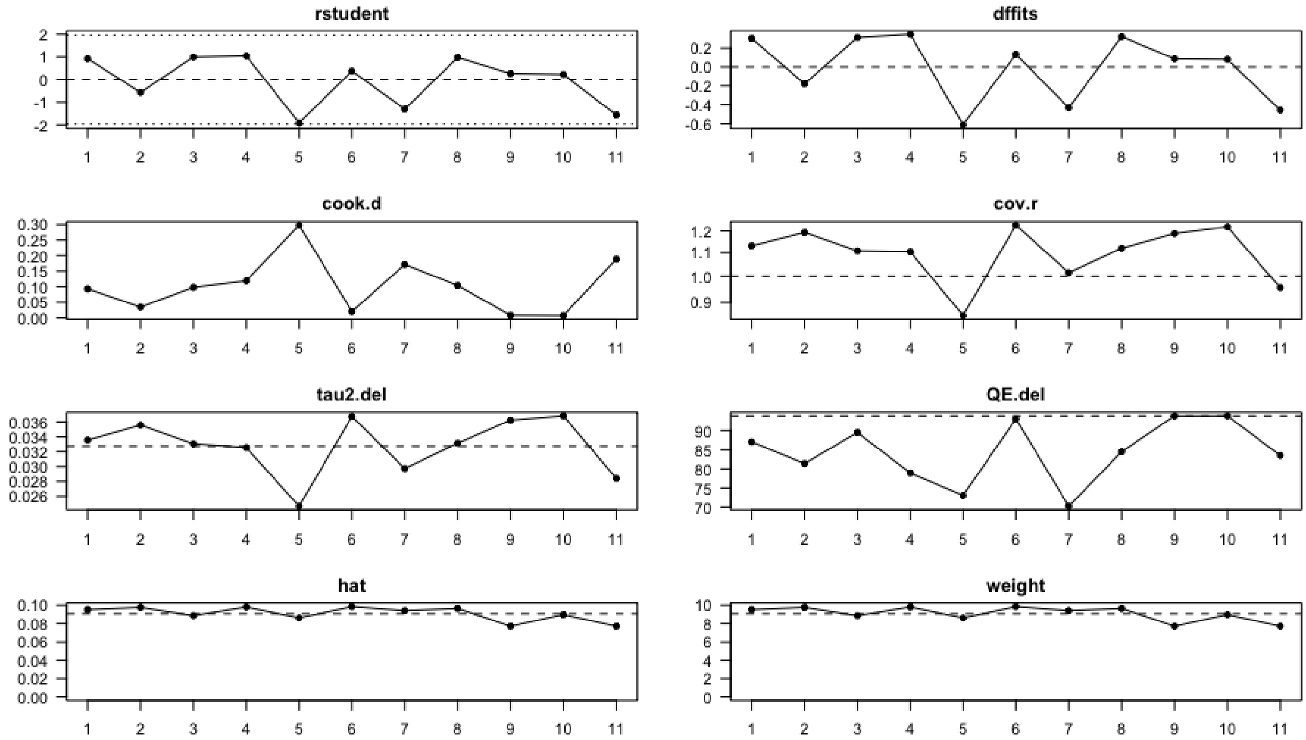

Figure S1: Plots showing influence measures of the included studies. (rstudent: Externally standardized residual; DFFITS: Difference in fits; cook.d: Cook's distances; cov.r: Covariance ratio; tau2.del: Leave-one-out amount of (residual) heterogeneity; QE.del: Leave-one-out test statistic of the test for (residual) heterogeneity; hat: Hat matrix)

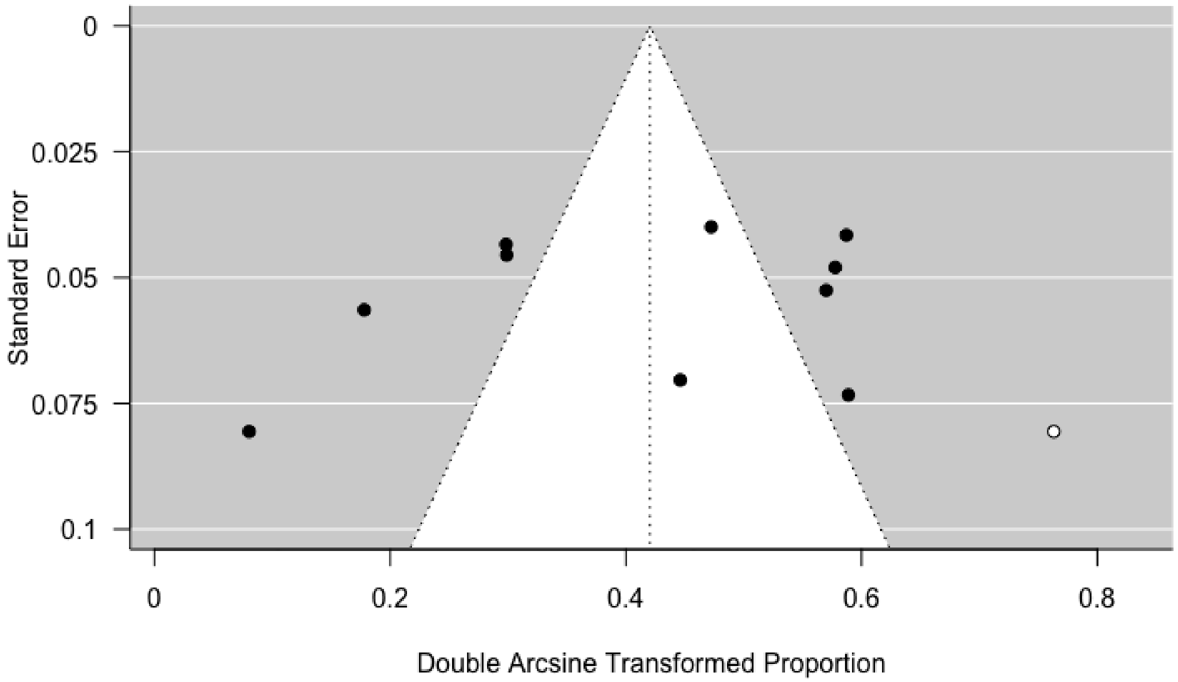

Figure S2: Trim and Fill Method for Meta-analysis conducted on all identified studies.

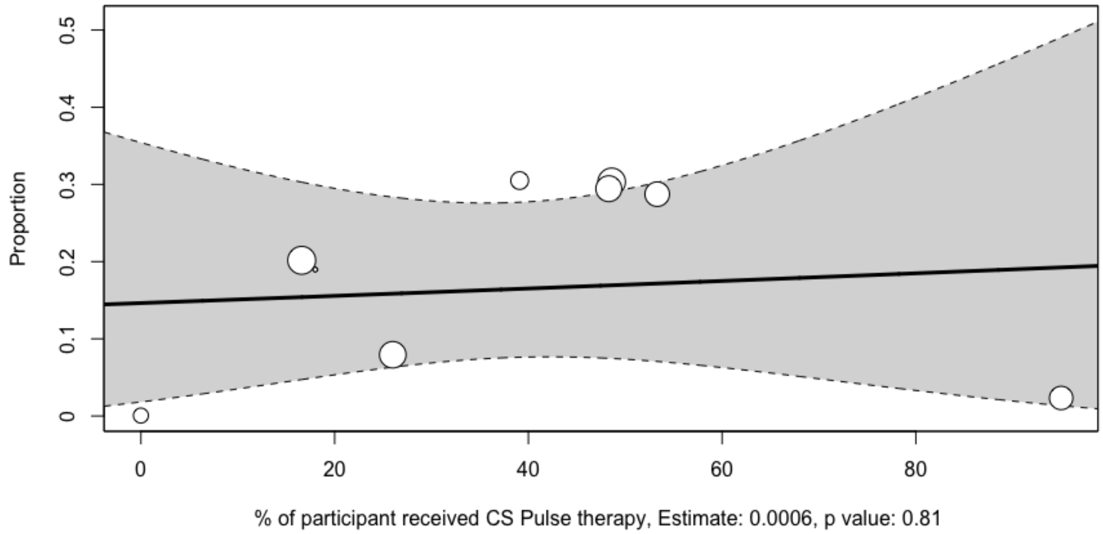

Figure S3: Meta-regression analysis conducted based on percent of participants received corticosteroid (CS) pulse therapy.

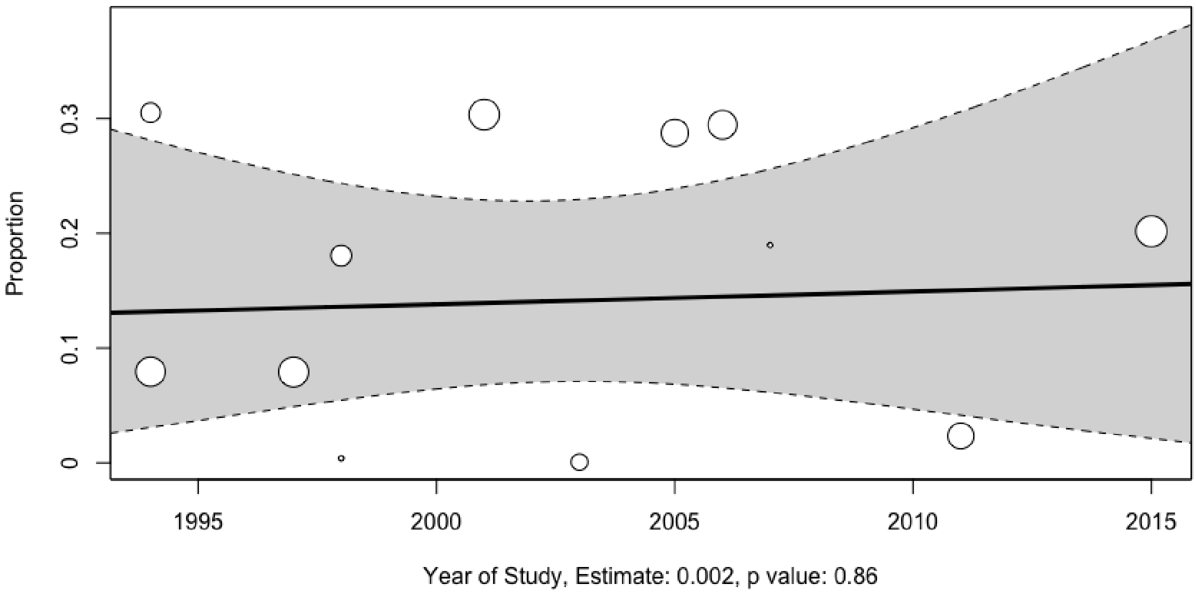

Figure S4: Meta-regression analyses conducted based on year of study.

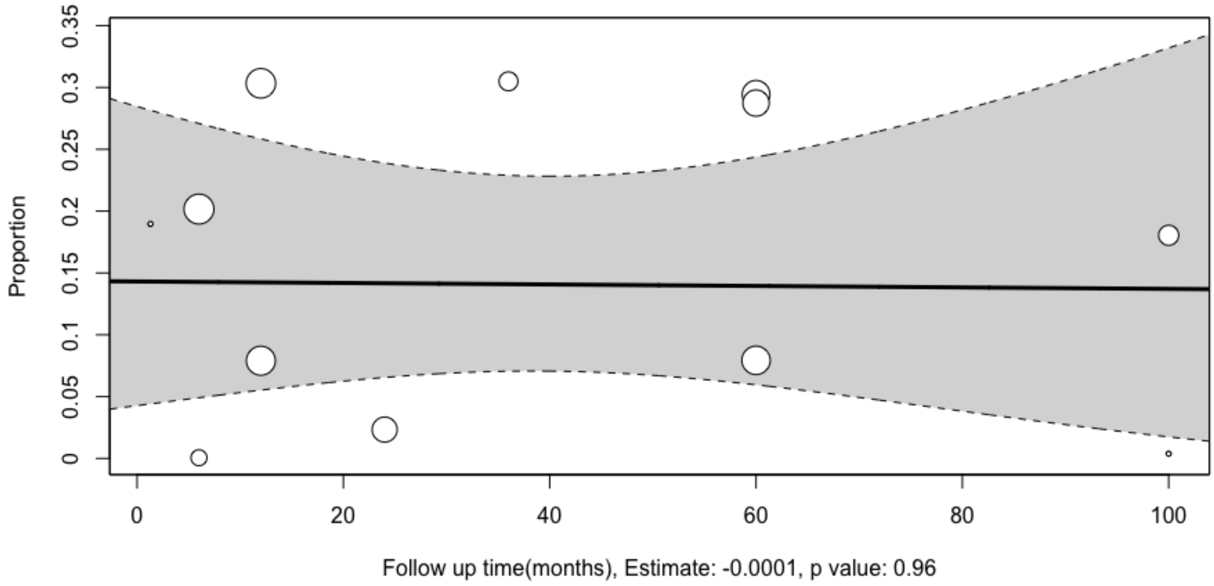

Figure S5: Meta-regression analyses conducted based on follow-up time.

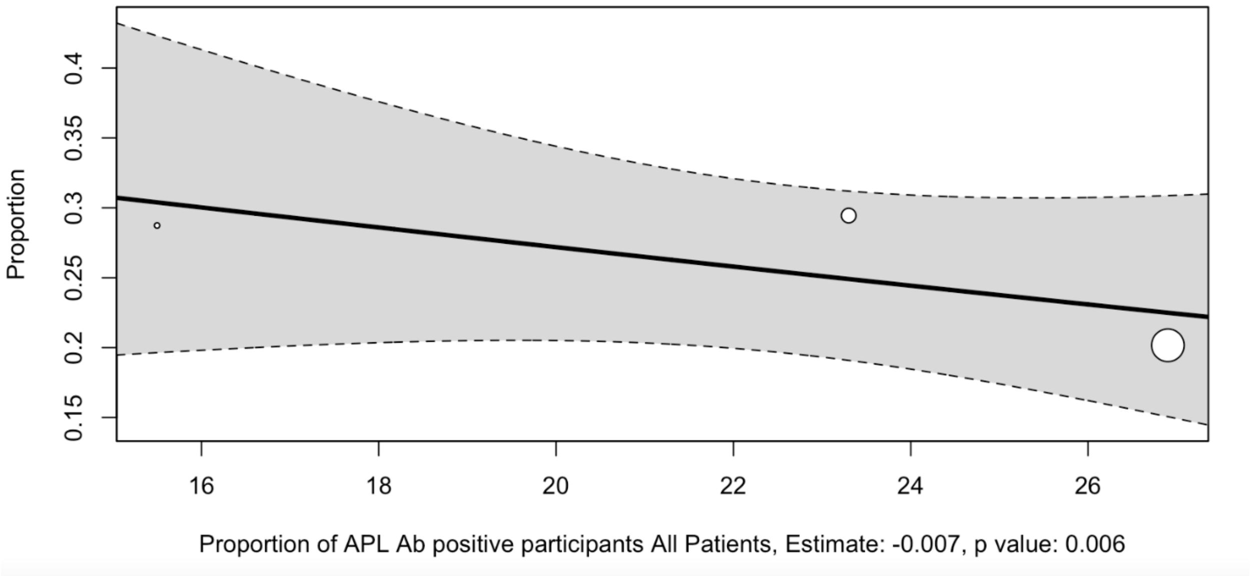

Figure S6: Meta-regression analyses conducted based on percent of participants with positive APL Antibody.

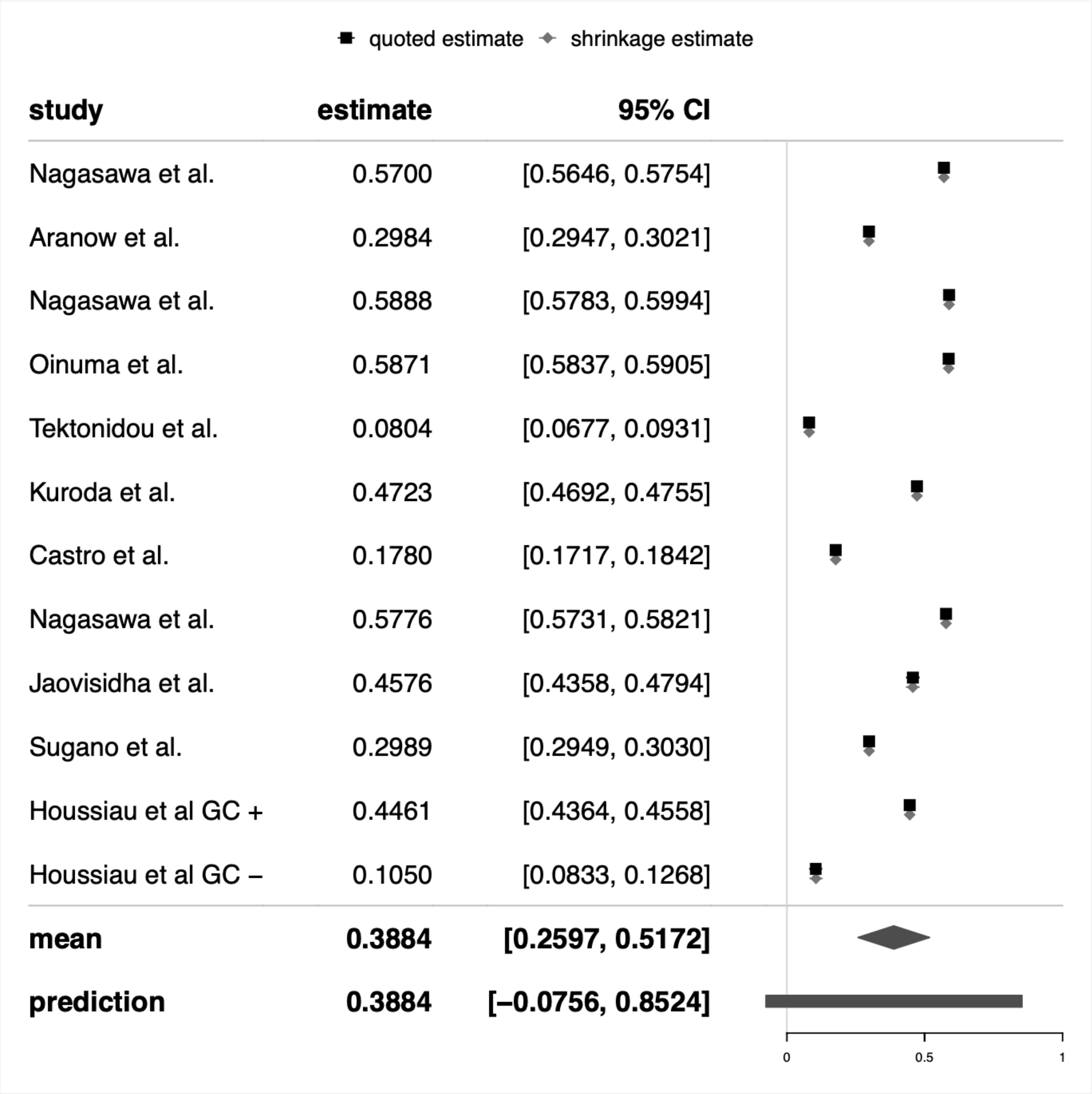

Figure S7: Bayesian Meta-analysis forest plot, All Participants

56. Jeong, H.-J., Kim, D., Cho, S.-K., Kim, Y., Bae, S.-C. and Sung, Y.-K. Clinical characteristics of multifocal osteonecrosis in Korean patients with rheumatic disease. *Int J Rheum Dis*. **2018**, *21*, 1301-1308.
57. Bogmat L.F; Bessonova I.N; Shevchenko N.S; Damage Index in Children with Systemic Lupus Erythematosus in 'Proceedings of the 25th European Paediatric Rheumatology Congress (PReS 2018)'. *Pediatr Rheumatol*. **2018,** *16* (Suppl 2), *52* (2018).
58. Klippel JH, Gerber LH, Pollak L, Decker JL. Avascular necrosis in systemic lupus erythematosus. Silent symmetric osteonecroses. *Am J Med*. **1979**, 67(1):83-87
59. Sekiya F.; Yamaji K; Yang K.; Tsuda H.; Takasaki Y. Investigation of occurrence of osteonecrosis of the femoral head after increasing corticosteroids in patients with recurring systemic lupus erythematosus. *Rheumatol Int*. **2010**, 30(12):1587-1593.

60. Sakamoto M.; Shimizu K.; Iida S.; Akita T.; Moriya H.; Nawata Y. Osteonecrosis of the femoral head: a prospective study with MRI. *J Bone Joint Surg Br*. **1997**, 79(2):213-219.
61. Fialho S.C.; Bonfá E.; Vitule L.F.; et al. Disease activity as a major risk factor for osteonecrosis in early systemic lupus erythematosus. *Lupus*. **2007**, 16(4):239-244.
62. Shigemura T.; Nakamura J.; Kishida S. et al. Incidence of osteonecrosis associated with corticosteroid therapy among different underlying diseases: prospective MRI study. *Rheumatology (Oxford)*. **2011**, 50(11):2023-2028.
63. Hurley R.M.; Steinberg R.H.; Patriquin H.; Drummond K.N.; A vascular necrosis of the femoral head in childhood systemic lupus erythematosus. *Can Med Assoc J*. **1974**, 111(8):781-784.
64. Chinnadurai S.; Chilukuri B.; Mahendran B.; Mantharam V.; Selvakumar B.; Sankaralingam R.; Clinical profile of osteonecrosis in systemic lupus erythematosus - Experience from a tertiary care centre in South India. *J Family Med Prim Care*. **2020**, 9(8):4363-4367.
65. Bergstein J.M.; Wiens C.; Fish A.J.; Vernier R.L.; Michael A.; Avascular necrosis of bone in systemic lupus erythematosus. *J Pediatr*. **1974**, 85(1):31-35.
66. Hagiwara S.; Nakamura J.; Watanabe A.; et al. Corticosteroids and low bone mineral density affect hip cartilage in systemic lupus erythematosus patients: Quantitative T2 mapping. *J Magn Reson Imaging*. **2015**;42(6):1524-1531
67. Lee J.; Kwok S.K.; Jung S.M.; et al. Osteonecrosis of the hip in Korean patients with systemic lupus erythematosus: risk factors and clinical outcome. *Lupus*. **2014**;23(1):39-45.
68. Nawata K.; Nakamura J.; Ikeda K.; et al. Transitional changes in the incidence of osteonecrosis in systemic lupus erythematosus patients: focus on immunosuppressant agents and glucocorticoids. *Rheumatology (Oxford)*. **2018**;57(5):844-849.
69. Nakamura J.; Saisu T.; Yamashita K.; Suzuki C.; Kamegaya M.; Takahashi K. Age at time of corticosteroid administration is a risk factor for osteonecrosis in pediatric patients with systemic lupus erythematosus: a prospective magnetic resonance imaging study [published correction appears in Arthritis Rheum. **2010** Nov;62(11):3248. Dosage error in article text]. *Arthritis Rheum*. **2010**;62(2):609-615.
70. Yamamoto S.; Watanabe A.; Nakamura J.; et al. Quantitative T2 mapping of femoral head cartilage in systemic lupus erythematosus patients with noncollapsed osteonecrosis of the femoral head associated with corticosteroid therapy. *J Magn Reson Imaging*. **2011**;34(5):1151-1158.
71. Oh S.N.; Jee W.H.; Cho S.M.; et al. Osteonecrosis in patients with systemic lupus erythematosus: MR imaging and scintigraphic evaluation. *Clin Imaging*. **2004**;28(4):305-309.
72. Nakamura J, Harada Y, Oinuma K, Iida S, Kishida S, Takahashi K. Spontaneous repair of asymptomatic osteonecrosis associated with corticosteroid therapy in systemic lupus erythematosus: 10-year minimum follow-up with MRI. *Lupus*. **2010**, 19(11):1307-1314.
73. Chen S.; Cai Q.; Xu Y. et al. Associations between glucocorticoids, antiphospholipid antibodies and femur head necrosis in patients with SLE: a directed acyclic graph-based multicentre study. *Ther Adv Musculoskelet Dis*. 2021;13:1759720X211002677. Published 2021 Mar 29.
